# Investigation of pulmonary artery and circulating endothelin-1 expression in dogs with pulmonary hypertension secondary to myxomatous mitral valve disease

**DOI:** 10.14202/vetworld.2024.2144-2151

**Published:** 2024-09-25

**Authors:** Nattawan Tangmahakul, Anudep Rungsipipat, Sirilak Disatian Surachetpong

**Affiliations:** 1 Department of Veterinary Medicine, Faculty of Veterinary Science, Chulalongkorn University, Henri-Dunant Road, Pathumwan, Bangkok 10330, Thailand; 2 Center of Excellence for Companion Animal Cancer (CE-CAC), Department of Pathology, Faculty of Veterinary Science, Chulalongkorn University, Henri-Dunant Road, Pathumwan, Bangkok 10330, Thailand

**Keywords:** canine, degenerative mitral valve disease, endothelin, post-capillary pulmonary hypertension

## Abstract

**Background and Aim:**

Pulmonary hypertension (PH) is a condition characterized by abnormally elevated pressure in the pulmonary vasculature. It is a common complication of myxomatous mitral valve disease (MMVD) in dogs. Several vasoactive substances, including endothelin-1 (ET-1), have been suggested to contribute to pathological changes in the pulmonary arteries of patients with PH. This study aimed to examine the local and systemic expression of ET-1 in dogs with PH secondary to MMVD.

**Materials and Methods:**

Lung tissues were collected from 20 client-owned dogs during the first stage of the study and divided into three groups: normal dogs (n = 5), MMVD dogs (n = 8), and MMVD+PH dogs (n = 7). The expression of ET-1 and endothelin A receptor (ET_A_R) in the pulmonary arteries was determined using immunohistochemistry. Blood samples were collected from 61 client-owned dogs for the second stage of the study and divided into three groups: normal (n = 22), MMVD (n = 20), and MMVD+PH (n = 19). Plasma ET-1 concentration was measured using a sandwich enzyme-linked immunosorbent assay.

**Results:**

There was no difference in ET-1 and ET_A_R expression in the pulmonary arteries among the three groups. Similarly, there was no difference in the plasma ET-1 concentration between the groups. In addition, no correlation was found between the immunohistochemical expression of ET-1 and ET_A_R and the thickness of the pulmonary arteries or between the plasma ET-1 level and echocardiographic variables.

**Conclusion:**

The lack of difference in the expression of ET-1 and ET_A_R in the pulmonary arteries and in the circulating ET-1 concentration among the studied groups suggests that ET-1 may not be related to the pathological development of PH secondary to MMVD in dogs. Due to the small sample size in this study, further research is needed to confirm these findings.

## Introduction

Pulmonary hypertension (PH) is characterized by abnormally elevated pulmonary vasculature pressure. It is classified into two types: pre-capillary and post-capillary, based on the underlying hemodynamic and echocardiographic findings [1, 2]. In dogs, post-capillary PH is the most common form and is often a complication of myxomatous mitral valve disease (MMVD) [3]. Dogs at advanced stages of MMVD are at higher risk of developing PH and have a shorter survival time [4, 5]. PH is associated with changes in the structure of the pulmonary artery, including hypertrophy and hyperplasia of the smooth muscle cell layers [6].

Endothelin-1 (ET-1) is a potent vasoconstrictor and a major component involved in the development of PH. It is synthesized primarily by endothelial cells [7, 8]. In the lungs, it is produced mainly by pulmonary artery endothelial cells (PAECs) and acts on smooth muscle cells below the endothelial layer in a paracrine manner [9]. ET-1 induces the proliferation and migration of human PAECs and activates intracellular calcium mobilization [10]. Prolonged or chronic activation of PAECs in patients with PH results in endothelial cell dysfunction and the release of vasoconstrictive factors, including ET-1 [11]. Pulmonary artery smooth muscle cells (PASMCs) can also synthesize ET-1 to promote their proliferation through an autocrine mechanism [12, 13]. ET-1 has been implicated in PH in human patients with and without congestive heart failure [14–18]. The endothelin A receptor (ET_A_R) plays proliferative and constrictive roles in PASMCs [19]. Polymorphisms of the ET-1 and ET_A_R genes have been found in patients with cardiovascular diseases, including systemic hypertension, coronary artery disease, and rheumatic mitral valve disease, which can lead to PH [20, 21]. Moreover, increased plasma ET-1 levels have been observed in patients with hypertension and atherosclerosis, potentially indicating a risk of heart failure [21, 22]. In dogs, elevated plasma ET-1 levels are also associated with pulmonary vascular remodeling in pre-capillary PH due to chronic thromboembolism [23].

To the best of our knowledge, no study has specifically investigated ET-1 expression in post-capillary PH resulting from left-sided heart disease, including MMVD. It would be interesting to examine the relationship between ET-1 and the development of post-capillary PH due to left-sided heart disease, particularly MMVD. This study investigated the involvement of local and circulating ET-1 in the development of PH in dogs with MMVD. This was performed by examining the expression of ET-1 and ET_A_R in the pulmonary arteries and plasma ET-1 concentrations. The results of this study may help clarify the role of ET-1 in the development of PH in dogs with MMVD.

## Materials and Methods

### Ethical approval and Informed consent

The animals were handled according to high ethical standards and national legislation. The study protocol, which involved blood collection and measurement of ET-1 concentration, was approved by the Institutional Animal Care and Use Committee, Faculty of Veterinary Science, Chulalongkorn University (approval number 1831053). Consent for blood collection was obtained from the dogs’ owners.

### Study period and location

The study was conducted from September 2021 to March 2023. Lung tissue samples were collected from the Pathology Division, Small Animal Teaching Hospital, Faculty of Veterinary Science, Chulalongkorn University, Bangkok, Thailand and processed at the Pathology Laboratory, Department of Veterinary Pathology, Faculty of Veterinary Science, Chulalongkorn University. Blood samples were collected from the Cardiology Clinic, Small Animal Teaching Hospital, Faculty of Veterinary Science, Chulalongkorn University, Bangkok, Thailand and processed at the Veterinary Diagnostic Laboratory, Faculty of Veterinary Science, Chulalongkorn University.

### The first stage: Investigation of ET-1 and endothelin-A receptor protein expression in pulmonary arteries

Lung tissue samples were collected from the cadavers of 20 small breeders. The dogs were 7–15 years old and weighed up to 10 kg. The samples were obtained during a necropsy at the Pathology Unit of the Small Animal Teaching Hospital, Faculty of Veterinary Science, Chulalongkorn University, Bangkok, Thailand. The dogs were divided into three groups: Healthy dogs (normal group), dogs with stage C–D MMVD (MMVD group), and dogs with PH secondary to stage C–D MMVD (MMVD+PH group). The normal group consisted of dogs that had died from causes unrelated to the cardiorespiratory system. Only dogs with stage C and D MMVD with or without PH were included in the MMVD and MMVD+PH groups, respectively. Dogs with other cardiovascular diseases and PH from other causes were excluded from the study. MMVD and PH were classified according to the American College of Veterinary Internal Medicine (ACVIM) consensus guidelines [2, 24]. Briefly, dogs with stage C MMVD had clinical signs of congestive heart failure with a left atrium (LA) to aorta (Ao) dimension ratio on the right parasternal short-axis view in early diastole ≥1.6 and a normalized value of the left ventricular internal diameter in end-diastole (LVIDdN) ≥1.7 cm/kg [24]. Intermediate and high probabilities of PH resulting from MMVD were determined by the peak echocardiographic velocity of tricuspid regurgitation (TR) and the number of anatomic sites of echocardiographic signs of PH. Intermediate probability of PH was defined as a peak TR velocity ≥3 m/s with or without anatomic sites of echocardiographic signs of PH in the right ventricle, pulmonary artery, right atrium, or caudal vena cava. A high probability of PH was defined by a peak TR velocity ≥3 m/s or >3.4 m/s with ≥2 or ≥1 anatomic sites of echocardiographic signs of PH, respectively [2].

The collected lung tissue samples were fixed in 10% neutral buffered formalin and embedded in paraffin. The tissue sections were then cut into a thickness of 4 μm. Immunohistochemical staining was performed using a standard method. Briefly, tissue sections were deparaffinized and rehydrated, pre-treated with 10 mM citrate buffer (pH 6.0) for 10 min in a high-power microwave oven, and washed with phosphate buffered saline (PBS). To inhibit endogenous peroxidase activity, tissues were treated with 10% hydrogen peroxide for 10 min and washed with PBS. Non-specific binding was blocked with 1% bovine serum albumin. Lung tissue sections were incubated with 1:200 dilutions of mouse monoclonal anti-ET-1 antibody (E166, Sigma-Aldrich, Missouri, USA) at 4°C overnight and 1:200 rabbit polyclonal anti-ET_A_R antibody (A405, Immuno-Biological Laboratories, Gunma, Japan) at 37°C for 1 h. Horseradish peroxidase-conjugated anti-rabbit/mouse secondary antibodies (EnVision Detection Systems, K5007, Dako, California, USA) were used for 45 min at 25°C. After washing with PBS, the ET-1 and ET_A_R reactions were visualized with 3,3’-diaminobenzidine tetrahydrochloride (EnVision Detection Systems, K5007, Dako, California, USA) and counterstained with Mayer’s hematoxylin. Ten pulmonary arteries with an external diameter of 200–400 μm were randomly selected and photographed under a photomicroscope (Eclipse Ci-L, Nikon, Kanagawa, Japan) at 100× magnification. The external and internal diameters of the photographed H&E-stained pulmonary arteries were measured using an image analyzer program (NIS-Elements Software, version 4.0, Nikon, Kanagawa, Japan). The percentage of a medial thickness (%MT) was calculated by dividing the difference between the external and internal diameters by the external diameter and multiplying by 100. The total areas and positive ET-1 and ET_A_R staining areas of the 10 pulmonary artery walls were captured and measured at 400× magnification and the percentage of positive staining areas was calculated by multiplying the ratio between the positive and total areas of the pulmonary arteries by 100.

### The second stage: Investigation of circulating ET-1

Sixty-one client-owned dogs aged 7–15 years and weighed up to 10 kg were presented to the Small Animal Teaching Hospital, Faculty of Veterinary Science, Chulalongkorn University, Bangkok, Thailand. History, physical examination, thoracic radiography, and electrocardiography were performed, and data were recorded. Dogs with systemic, infectious, inflammatory, or other cardiorespiratory diseases that could lead to PH were excluded from the study. The enrolled dogs were divided into three groups: 22 healthy dogs (normal group), 20 dogs with stage C MMVD (MMVD group), and 19 dogs with PH secondary to MMVD (MMVD+PH group). An experienced cardiologist performed an echocardiographic examination on all dogs using a 4–12 MHz phased array transducer on an ultrasound machine (M9, Mindray, Shenzhen, China). MMVD staging and assessment of PH probability were carried out according to the ACVIM consensus guidelines [2, 24]. The normal group consisted of healthy dogs visiting for health checkups who were confirmed to have no cardiorespiratory abnormalities or diseases.

Two milliliters of whole blood samples were collected from the enrolled dogs through venipuncture of the cephalic or lateral saphenous veins. Blood samples were centrifuged at 1500× *g* for 20 min to collect plasma (High Speed Refrigerated Micro Centrifuge MX-307, Tomy, Tokyo, Japan) and stored at –80°C until processing. A commercial sandwich enzyme-linked immunosorbent assay (ELISA) kit for ET-1 (Dog Canine Endothelin 1/EDN1 ELISA kit PicoKine™, Boster Biological Technology, California, USA) was used to measure the ET-1 concentration. The optical density of all plasma samples was measured at a wavelength of 450 nm using a microplate spectrophotometer (Epoch 2 microplate reader, BioTek, California, USA). Intra-assay and inter-assay precision and linearity of dilution were analyzed as part of the in-house ELISA validation.

### Statistical analysis

Data were statistically analyzed using SPSS version 22 (IBM Corp., NY, USA). The distribution of data was assessed using the Shapiro–Wilk test. The percentage of positive areas of ET-1 and ET_A_R expression was expressed as mean ± standard deviation (SD) and analyzed by one-way analysis of variance. The correlation between the expression of ET-1 and ET_A_R and variables, including age, sex, body weight, and echocardiographic data, was analyzed using Pearson’s correlation coefficient. Plasma ET-1 concentration was expressed as median and interquartile range and analyzed using the Kruskal–Wallis test. Multiple linear regression was used to control for potential confounders, including age and gender. The correlation between plasma ET-1 concentration and variables, including age, sex, body weight, and echocardiographic data, was analyzed using Spearman’s correlation. Statistical significance was set at p < 0.05.

## Results

### Investigation of ET-1 and endothelin-A receptor protein expression in pulmonary arteries

The normal group (n = 5) consisted of three males and two females (three Shih Tzus, one Cocker Spaniel, and one Pomeranian). The MMVD group (n = 8) included two males and six females (four Poodles, one Yorkshire Terrier, one Shih Tzu, one Pomeranian, and one mixed breed dog). The MMVD+PH group (n = 7) comprised three males and four females (two Chihuahuas, one Shih Tzu, one Poodle, one Pomeranian, one Schnauzer, and one mixed breed dog). There were no differences in age or body weight between the groups. Six dogs with stage C MMVD and two dogs with stage D MMVD were included in the MMVD group. Four dogs had stage C MMVD and three dogs had stage D MMVD, along with low (n = 2), intermediate (n = 1), and high probability of PH (n = 4). MMVD and MMVD+PH dogs were treated with standard medications, including angiotensin-converting enzyme inhibitors (ACEIs) (n = 7 and n = 7), furosemide (n = 7 and n = 7), pimobendan (n = 7 and n = 7), spironolactone (n = 2 and n = 4), and a combination of amiloride and hydrochlorothiazide (n = 1 and n = 2). One dog in the MMVD+PH group with stage D MMVD and a high probability of PH was treated with sildenafil. There were no significant differences in echocardiographic data between dogs with MMVD and those with MMVD+PH. The demographic and echocardiographic characteristics of the enrolled dogs are presented in Table 1.

**Table-1:** Signalment, clinical characteristics, echocardiographic data, and immunohistochemical ET-1 and ET_A_R expression of the dogs.

Parameter	Normal (n = 5)	MMVD (n = 8)	MMVD+PH (n = 7)	p-value
Age (year)	9.60 ± 3.85	13.50 ± 2.45	13.29 ± 2.56	0.062
Gender (M/F)	3/2	2/6	3/4	-
Body weight (kg)	5.40 ± 0.32	4.08 ± 1.57	4.87 ± 2.14	0.363
Stage C/D MMVD	-	6/2	4/3	-
LA (cm/kg)	-	1.79 ± 0.42	2.42 ± 0.62	0.057
Ao (cm/kg)	-	0.94 ± 0.22	1.19 ± 0.20	0.062
LA/Ao	-	1.94 ± 0.42	2.09 ± 0.73	0.649
IVSdN (cm/kg)	-	0.62 ± 0.22	0.69 ± 0.18	0.577
LVIDdN (cm/kg)	-	2.38 ± 0.67	2.60 ± 0.69	0.580
LVPWdN (cm/kg)	-	0.52 ± 0.18	0.65 ± 0.22	0.256
IVSsN (cm/kg)	-	0.93 ± 0.31	0.87 ± 0.42	0.804
LVIDsN (cm/kg)	-	1.25 ± 0.27	1.27 ± 0.63	0.958
LVPWsN (cm/kg)	-	0.80 ± 0.23	0.99 ± 0.35	0.289
%FS	-	45.11 ± 9.56	52.06 ± 13.56	0.317
Peak TR velocity (m/s)	-	-	3.80 ± 0.88	-
ET-1 positive area (%)	38.79 ± 14.96	34.33 ± 14.37	42.89 ± 9.32	0.459
ET_A_R-positive area (%)	24.73 ± 12.11	25.70 ± 10.87	33.57 ± 10.21	0.298

Ao=Aorta, ET_A_R=Endothelin A receptor, ET-1=Endothelin-1, FS=Fractional shortening, IVSdN=Normalized interventricular septal thickness at end-diastole, IVSsN=Normalized interventricular septal thickness at end-systole, LA=Left atrium, LA/Ao=Left atrium-to-aorta ratio, LVIDdN=Normalized left ventricular internal diameter at end-diastole, LVIDsN=Normalized left ventricular internal diameter at end-systole, LVPWdN=Normalized left ventricular posterior wall thickness at end-diastole, LVPWsN=Normalized left ventricular posterior wall thickness at end-systole, TR=Tricuspid regurgitation

**Figure-1:**
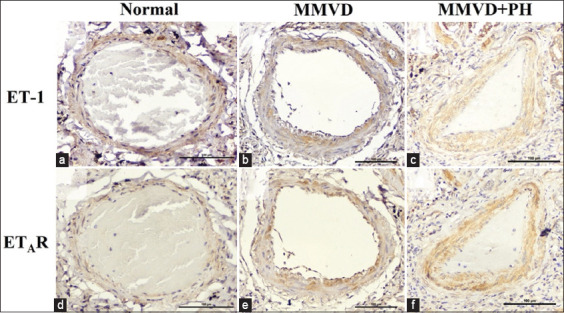
Histopathological findings and ET-1 and ET_A_R expression in the pulmonary artery wall of the Normal (a and d), MMVD (b and e), and MMVD+PH (c and f) groups. Both ET-1 and ETAR expressions were found as brown staining in the smooth muscle layer of the pulmonary arterial wall. (Labeled streptavidin-biotin, Immunohistochemistry, Mayer’s Hematoxylin counterstained, 400× magnification). ET-1=Endothelin-1, ET_A_R=Endothelin A receptor, PH=Pulmonary hypertension, MMVD=Myxomatous mitral valve disease.

**Figure-2:**
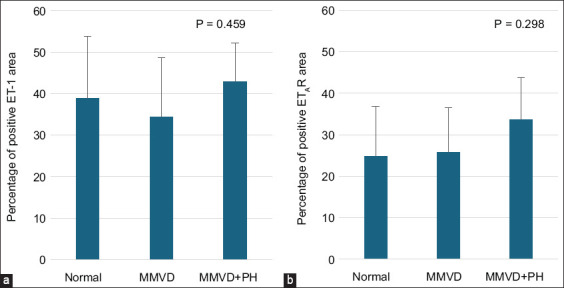
Percentage of positive areas for ET-1 (a) and ET_A_R (b) in the Normal (n = 5), MMVD (n = 8) and MMVD+PH (n = 7) groups. The data were presented as mean and standard deviation (error bar). ET-1=Endothelin-1, ET_A_R=Endothelin A receptor, PH=Pulmonary hypertension, MMVD=Myxomatous mitral valve disease.

Immunohistochemistry showed that the expression of ET-1 and ET_A_R was presented in the cytoplasm of PASMCs and PAECs in all groups (Figure-1). The expression of ET-1 in the pulmonary arteries did not differ between the groups (Table-1 and Figure-2a). Although the expression of ET_A_R tended to be upregulated in the MMVD+PH group, there was no significant difference in the percentage of positive ET_A_R staining area between the groups, indicating that ET_A_R expression did not change in dogs with MMVD+PH compared with the other groups (Table-1 and Figure-2b). The medial thickness of the pulmonary arteries of the dogs included in the study ranged from 200 to 400 μm. The %MT of the pulmonary arteries was not correlated with the expression of ET-1 or ET_A_R. However, the expression of ET-1 was strongly correlated with ET_A_R expression (r = 0.716, p < 0.0001). There was no correlation between expression and echocardiographic parameters.

### Investigation of circulating ET-1

The dogs in the normal group (n = 22) included eight males and 14 females, which comprised eight Shih Tzus, five Chihuahuas, five Yorkshire Terriers, one Dachshund, and three mixed-breed dogs. The MMVD group (n = 20) consisted of 15 males and five females, including five Chihuahuas, five Pomeranians, four Poodles, one Beagle, one Chinese Crested Dog, one Finnish Spitz, one Shih Tzu, one Yorkshire Terrier, and one mixed breed dog. The MMVD+PH group (n = 19) comprised eight males and 11 female dogs, including six Poodles, four Chihuahuas, two Miniature Pinschers, two Shih Tzus, one Jack Russel Terrier, and four mixed-breed dogs. The age of the healthy dogs was significantly younger than that of the MMVD and MMVD+PH dogs (p < 0.001). All dogs in the MMVD and MMVD+PH groups had stage C MMVD with increased LA/Ao and LVIDdN compared with the normal group (p < 0.001). Dogs in the MMVD+PH group had intermediate (n = 10) and high probability of PH (n = 9). In the MMVD and MMVD+PH groups, dogs were treated with angiotensin-converting enzyme inhibitors (n = 16 and n = 18), furosemide (n = 20 and n = 19), pimobendan (n = 20 and n = 19), spironolactone (n = 5 and n = 4), or a combination of amiloride and hydrochlorothiazide (n = 3 and n = 3). One dog in the MMVD group was treated with digoxin. Four MMVD dogs with PH were treated with sildenafil. The signalment and echocardiographic data are presented in Table 2.

**Figure-3:**
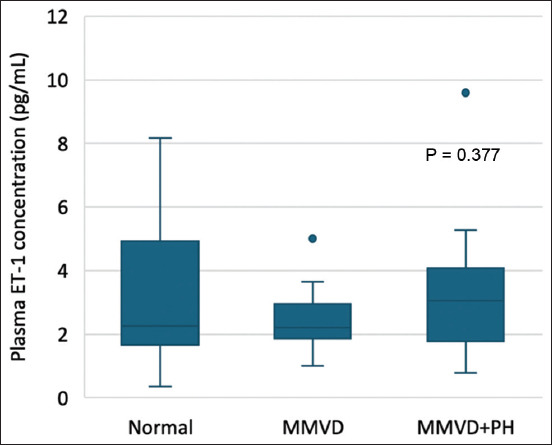
Box and Whisker plots of the ET-1 concentration in the plasma of normal dogs (n = 22), MMVD dogs (n = 20), and MMVD+PH dogs (n = 19). The median plasma ET-1 concentration is expressed as the horizontal lines of the boxes. The first and third quartiles are represented as the lower and upper ends, respectively, of the boxes. The minimum and maximum values of the data are shown at the ends of the whisker. The circles represent data outliers. ET-1=Endothelin-1, MMVD=Myxomatous mitral valve disease, PH=Pulmonary hypertension.

Comparison of plasma ET-1 concentrations revealed no significant differences among the normal, MMVD, and MMVD+PH groups (Table-2 and Figure-3). After accounting for confounding factors, including age and sex, the plasma ET-1 concentration did not differ significantly between the groups (p = 0.197). The plasma ET-1 concentration was weakly correlated with fractional shortening (r = 0.271, p = 0.034). There was no difference in plasma ET-1 concentration between male (2.32 [1.51–3.66] pg/mL) and female dogs (2.33 [1.83–3.42] pg/mL) in the present study. In-house ELISA validation tests revealed that the intra-assay precision, inter-assay precision, and linearity of dilution were 5.41%, 8.60%, and 105.10%, respectively.

**Table-2:** Signalment, clinical characteristics, echocardiographic data, and plasma ET-1 concentrations of the dogs included in this study.

Parameter	Normal (n = 22)	MMVD (n = 20)	MMVD+PH (n = 19)	p-value
Age (year)	8.00 (7.00–10.00)	12.00 (11.00–13.75)^a^	12.00 (10.00–14.00)^a^	<0.001
Gender (M/F)	8/14	15/5	8/11	–
Body weight (kg)	4.65 (2.83–6.01)	5.16 (3.76–6.53)	5.22 (4.60–6.32)	0.147
LA (cm/kg)	1.00 (0.91–1.13)	2.23 (1.88–2.60)^a^	1.65 (1.37–2.48)^a^	<0.001
Ao (cm/kg)	0.78 (0.68–0.92)	1.18 (0.92–1.36)^a^	0.78 (0.61–1.13)^b^	0.007
LA/Ao	1.27 (1.13–1.41)	1.82 (1.58–2.36)^a^	2.18 (2.01–2.24)^a^	<0.001
IVSdN (cm/kg)	0.45 (0.40–0.49))	0.47 (0.40–0.50)	0.45 (0.40–0.54)	0.774
LVIDdN (cm/kg)	1.27 (1.20–1.40)	1.86 (1.67–2.00)^a^	1.80 (1.71–2.00)^a^	<0.001
LVPWdN (cm/kg)	0.37 (0.32–0.43)	0.37 (0.33–0.43)	0.40 (0.35–0.43)	0.847
IVSsN (cm/kg)	0.59 (0.50–0.63)	0.56 (0.51–0.67)	0.62 (0.56–0.72)	0.217
LVIDsN (cm/kg)	0.75 (0.72–0.89)	1.02 (0.84–1.10)^a^	0.78 (0.67–1.13)	0.008
LVPWsN (cm/kg)	0.64 (0.55–0.68)	0.66 (0.60–0.77)	0.70 (0.57–0.78)	0.281
%FS	38.52 (33.91–44.73)	44.15 (38.38–48.96)	50.61 (43.23–55.74)^a^	0.002
Peak TR velocity (m/s)	–	–	3.72 (3.35–4.12)	–
Plasma ET–1 concentration (pg/mL)	2.26 (1.66–4.93)	2.21 (1.86–2.95)	3.05 (1.78–4.08)	0.377

Ao=Aorta, ET-1=Endothelin-1, FS=Fractional shortening, IVSdN=Normalized interventricular septal thickness at end-diastole, IVSsN=Normalized interventricular septal thickness at end-systole, LA=Left atrium, LA/Ao=Left atrium-to-aorta ratio, LVIDdN=Normalized left ventricular internal diameter at end-diastole, LVIDsN=Normalized left ventricular internal diameter at end-systole, LVPWdN=Normalized left ventricular posterior wall thickness at end-diastole, LVPWsN=Normalized left ventricular posterior wall thickness at end-systole, TR=Tricuspid regurgitation. aindicates

a significant difference compared with the normal group (p < 0.05). ^b^indicates significant difference compared with the MMVD group (p < 0.05)

## Discussion

Endothelin, a protein involved in regulating vascular tone and resistance, has been identified as a key mediator of pulmonary arterial hypertension (PAH), and endothelin receptor antagonists are commonly used to treat this condition [25]. However, the relationship between endothelin and other forms of PH is not well understood. This study investigated the relationship between the endothelial system and PH secondary to MMVD, which is the most common cause of PH in veterinary medicine. The results of this study, however, did not find an association between the endothelin system and PH due to MMVD in dogs, as investigated in both tissue and circulation.

Sakarin *et al*. [6] has shown that increased MT can occur in dogs with MMVD both with and without PH, suggesting that pulmonary artery thickening may precede the development of post-capillary PH in these dogs. In human patients with PH, ET-1 expression has been found to be elevated in PAECs, with higher levels observed in primary PH compared with PH caused by other factors [14]. In a canine model of chronic embolic PH, increased ET-1 expression was observed in both endothelial and smooth muscle cells of the pulmonary arteries, along with elevated plasma ET-1 concentrations [23]. This evidence indicates that both the expression of ET-1 in the pulmonary arteries and the circulating levels of ET-1 are increased in patients with primary PAH or pre-capillary PH. This study found ET-1 and its receptor ET_A_R in the cytoplasm of PASMCs and PAECs in all groups. Although ET-1 and ET_A_R tended to increase in MMVD dogs with PH, no significant differences were found between the groups. In addition, this study did not identify a relationship between ET-1 and ET_A_R and pulmonary artery remodeling. This suggests that the expression of ET-1 and ET_A_R may differ in post-capillary and pre-capillary PH. A previous study by Tabeling *et al*. [26] indicated that the endothelin B receptor (ET_B_R) has an anti-inflammatory effect on the pathology of PAH, and ET_B_R deficiency may be associated with perivascular inflammation in the lungs. Therefore, further studies on PH in dogs should investigate the role of ET_B_R in the lungs.

The study included age-matched dogs, but the ages of the healthy dogs were lower than those of the dogs in the other disease groups. Despite this, the lack of a correlation between age and ET-1 concentration suggested that age did not affect the circulating ET-1 concentration. In addition, this study did not match the dogs by gender. However, there were no significant differences in plasma ET-1 levels between males and females, indicating that sex did not affect circulating ET-1 concentrations. After accounting for potential confounders, including age and sex, plasma ET-1 concentrations remained similar between groups. Thus, age and gender were not confounding factors for plasma ET-1 concentration in this study.

It is important to note that ET-1 produced by various cells, including vascular smooth muscle cells, cardiomyocytes, cardiac fibroblasts, polymorphonuclear leukocytes, and macrophages [12, 27–30], can be elevated for several reasons. Increased plasma ET-1 levels have been observed in dogs with congestive heart failure due to MMVD and dilated cardiomyopathy [31], and higher ET-1 levels have been associated with PH in humans with chronic congestive heart failure [15]. However, another study found no difference in serum ET-1 concentrations between patients with PH associated with rheumatic mitral valve disease and healthy controls [20]. We found a weak correlation between plasma ET-1 concentration and fractional shortening, likely because ET-1 affects ventricular myocardial contraction [32]. These results suggest the potential of ET-1 as a candidate biomarker for MMVD and PH in dogs. However, plasma ET-1 levels did not differ between normal dogs and those with other diseases. In this study, ET-1 levels tended to increase in dogs with MMVD and PH, but there were no significant differences between the groups. This finding is similar to that of a previous study by Oleynikov and Yi [33] in dogs, which reported higher ET-1 levels in dogs with pre-capillary PH but not in those with post-capillary PH. The difference in ET-1 levels between pre- and post-capillary PH suggests that ET-1 may be a useful marker for differentiating between the two types of PH in dogs [33]. To determine whether plasma ET-1 is a candidate biomarker for MMVD or post-capillary PH in dogs, larger sample sizes are needed for further studies.

An increase in ET-1 can result in either increased production or decreased clearance [16, 34]. Previous studies by Kim *et al*. [23] and Meoli *et al*. [35] in humans and experimental dogs have shown decreased ET-1 clearance in subjects with pre-capillary PH. In this study, histopathological analysis did not reveal increased ET-1 production in the pulmonary arteries. However, ET-1 clearance in post-capillary PHs remains unknown and requires further investigation.

Previous studies by Sakarin *et al*. [6] and Tangmahakul *et al*. [36] in dogs reported the relationship between the serotonin pathway and post-capillary PH. There is evidence suggesting an association between serotonin and endothelin in humans and other species [26], but no studies have specifically investigated this relationship in dogs with post-capillary PH.

## Conclusion

The expression of ET-1 and its receptors in the pulmonary arteries, as well as the plasma concentration of ET-1, did not differ between dogs with MMVD and PH and those with normal conditions or MMVD without PH. This suggests that ET-1 is not a significant factor in the development of post-capillary PH secondary to MMVD in dogs. This conclusion was based on a single study, and one limitation was the small sample size, which may have influenced the statistical significance of the results. To increase the precision of the findings and better represent the canine population, further studies should include a larger number of dog samples to confirm the relationship between ET-1 and post-capillary PH in dogs.

## Authors’ Contributions

NT: Conceptualization and study design, sample curation, conducted experiments, data analysis, and drafted the manuscript. AR: Conceptualization and study design, data analysis, and edited the manuscript. SDS: Conceptualization and study design, data analysis, supervision, and edited the manuscript. All authors have read, reviewed, and approved the final manuscript.
